# Variations and Asymmetry in Sacral Ventral Rami Contributions to the Bladder

**DOI:** 10.3390/diagnostics15010102

**Published:** 2025-01-03

**Authors:** Rebeccah R. Overton, Istvan P. Tamas, Emily P. Day, Nagat Frara, Michel A. Pontari, Susan B. Fecho, Steven N. Popoff, Mary F. Barbe

**Affiliations:** 1 Aging + Cardiovascular Discovery Center, Department of Biomedical Education and Data Science, Lewis Katz School of Medicine of Temple University, Philadelphia, PA 19140, USA; rebeccah.overton@temple.edu (R.R.O.); istvan.tamas@temple.edu (I.P.T.); nagat.frara@temple.edu (N.F.); 2 Department of Medical Education, Drexel University College of Medicine, Philadelphia, PA 19129, USA; epd45@drexel.edu; 3 Department of Urology, Lewis Katz School of Medicine of Temple University, Philadelphia, PA 19140, USA; michel.pontari@tuhs.temple.edu; 4 Department of Art, Barton College, Wilson, NC 27893, USA; sfecho@barton.edu; 5 Department of Biomedical Education and Data Science, Lewis Katz School of Medicine of Temple University, Philadelphia, PA 19140, USA; steven.popoff@temple.edu

**Keywords:** sacral plexus, bladder, vesical branches, ureter, neuromodulation

## Abstract

**Background/Objectives:** We have demonstrated in human cadavers and canines that nerve transfer to bladder vesical nerve branches is technically feasible for bladder reinnervation after nerve injury. We further clarify here that sacral (S) ventral rami contribute to these vesical branches in 36 pelvic sides (in 22 human cadavers). **Methods:** Gross post-mortem visualization and open anterior abdominal approaches were used, as was micro-CT of sacral nerve bundles, for further confirmation when needed. **Results:** Considerable between and within-subject variation was observed. Sacral (S) ventral rami contributions to vesical nerves were observed as shared contributions from several rami or, in a few cases, from single rami: S2 alone (6%), S3 alone (6%), S2 and S3 (28%), S3 and S4 (28%), S2–S4, 14%, L5 in combination with S1–S4 (6%), S1 and S2 (6%), and S3–S5 (3%). The most common contributor to these shared or single rami contributions was from the S3 ventral ramus, which contributed 100% of the time on the left side and 79% on the right side. Side-to-side asymmetry was observed in 10 of 14 cadavers examined bilaterally (71%). **Conclusions:** This characterization of the anatomical variation in sacral ventral rami contributions to the bladder will ultimately aid in developing therapeutics for patients with bladder dysfunction.

## 1. Introduction

There are estimated to be 305,000 people living with tSCI in the U.S., with less than 1% of patients making a complete neurological recovery at the end of their rehabilitation stay [1,2]. Traumatic spinal cord injuries are life-altering and often impact an individual’s ability to walk, work, and perform other necessary daily activities, such as voluntary bladder and bowel emptying [2,3,4,5]. Urinary dysfunction can also occur after surgical procedures for tethered cord, colorectal cancer, neurofibromas, and sacral tumors, with surgeries for detrusor hyperreflexia or after prostatic/gynecological surgeries [6,7]. Prolapsed discs in the L5/S1 intervertebral disc regions can also result in urinary dysfunction [8]. Loss of bladder control may further reduce an individual’s independence with untold impacts on their physical and mental health.

Regaining bladder function remains the top priority for patients [9]. As a result, studies have been investigating the ability to restore bladder function after spinal cord or root injury or damage of pelvic nerves. This has been attempted in several ways, including the use of somatic nerve transfer that seeks to bypass regions with injury, such as somatic–autonomic reflex pathway procedures in which a lumbar root is transferred to sacral roots innervating bladder in humans [10,11,12]. Sacral neurostimulation/modulation procedures are now common means for modulating complaints of bladder frequency and urgency [13,14,15].

We have recently reported variation in vesical nerve branching patterns to the bladder [16]. Yet, there are few studies in the current literature documenting which sacral root ventral rami sends direct contributions to the bladder wall in humans. One retrospective study examined 40 human subjects with complete paraplegia or partial tetraplegia in which sacral roots were electrically stimulated using a tripolar electrode, with sacral root contribution was measured using cystamanometry to identify bladder pressure and muscular contraction. It was found in that study that the most common contributor was from S3 (100%), with S2 following (60%), although many common combinations of sacral contributions included: S3–S4 (30%), S2–S3 (15%), S2–S4 (12.5%), and S3–S5 (5%) [7]. Side-to-side asymmetry in the response was observed in 7 of the 40 cases in this study (18%). Another study identified five patients with hypertonic neurogenic bladders and attempted to identify the motor fibers supplying the bladder detrusor muscle [17]. This was performed with selective sacral root rhizotomy and sacral root electrical stimulation using a Physio-Stimulator; pressures were measured using a cystometrogram. They found that S3 was the most dominant contributor to bladder contraction in all five patients, with some contribution from S4. Stimulation of S2 revealed variable results—in some patients, it caused leg contraction, while in others, it caused bladder contraction during stimulation of the most medial portion of the root. In all cases, when the medially located fibers of the roots were stimulated, the largest contractile forces and pressures were produced in the bladder [17]. In a third study, the bladder contractility response to neurostimulation was examined in two patients in which S2 and S3 nerve roots were stimulated before and after selective neurotomy or neural blockade [18]. Variability was found with one patient of these patients; stimulation of the left S3 ventral root produced a strong bladder contraction (80 cm water) with no urethral sphincter response. In contrast, stimulation of their right side produced a weak bladder response with a strong urethral sphincter pressure.

Even fewer studies have characterized the sacral contributions to the bladder using cadaveric dissection techniques. Several older studies (published between 1913 and 1934) show a range of primary contributions from either S2–S4 [19], S2–S3 [20], S2–S4 (and S1 in one cadaver) [21], or S3, with S2 and S4 giving additional nerve fibers [22]. There are two newer studies cadaveric studies on this topic [23,24]. Aurore and colleagues determined in 11 embalmed female pelves that pelvic splanchnics (the target organ was not identified) originated from S2–S4 primarily, while 18% of the cadavers showed an additional pelvic splanchnic nerve originating from the S1 ventral ramus [23]. In a similar study on the origin of splanchnic nerves in the pelvic region (again, no target organ identified), the examination of 85 hemipelves of approximately 63 cadavers showed that pelvic splanchnics arose primarily from S3 and S4 ventral rami (52% and 32%, respectively, in females; 60% and 37% in males) [24]. Lesser contributors to the pelvic splanchnics originated from S2 (12%) and S5 (4%) in females and S2 (4%) in males [24].

Although the number of studies cited above is few, they suggest variability between and within humans regarding sacral root contributions to the bladder, as well as side-to-side variability, although the extent of the segmental and side-to-side variabilities are unclear. For best preservation and restoration of bladder function during surgeries, improved anatomical knowledge of the existence of sacral root variability (i.e., a knowledge of normal individual variations [25]) will avoid damage to roots needed for bladder emptying, especially in patients who already have considerable functional losses [7,10,26,27]. Also, it has been suggested that the best outcomes of neurostimulation need to take into account individual variations—requiring full evaluation of all sacral root contributions rather than preferentially choosing S3, for example [7].

Thus, our objective was to examine which sacral ventral rami contributes nerve branches to the bladder in human cadavers and the extent of asymmetrical sidedness. We hypothesize that S3 will be the primary contributor of innervation to the bladder, although based on the literature, we anticipate that it will not be the only spinal nerve contributor.

## 2. Materials and Methods

### 2.1. Study Material 

Thirty-six pelvic sides were dissected in 22 human cadavers (11 females and 11 males). The age range of the cadavers in which the age was known was 67 to >90 (See Table S1). All cadavers were obtained from the Human Gift Registry program at Lewis Katz School of Medicine, Temple University, Department of Biomedical Education, Philadelphia. Our studies were performed in compliance with the policies of this institution.

### 2.2. Open Anterior Abdominal Approach

Twenty cadavers had been embalmed with formalin-phenol fixative and were studied as an extension of routine anatomical dissection courses: most required additional dissection. Cadavers were inspected to ensure that pelvic floor anatomy and bladder were intact. In cases where needed structures were damaged, structures were not assessed on 8 of 36 sides, see Table 1 and Table S1. Seven of these 20 cadavers were hemisected prior to our examinations; seven others were hemisected during the study (after our initial investigations while still intact) to aid in the identification of pelvic structures. Six of the embalmed cadavers were left intact (not hemisected) (Table S1). The hemisections also allowed examination of the posterior pelvic region for the identification of ventral rami contributions to the bladder. Vesical branches of the inferior hypogastric plexus were located on the posterolateral aspect of the bladder wall and were identified and traced back to their respective sacral roots. The sacral vertebral, sacral promontory, pubis, ureter and obturator nerves were used as landmarks.

### 2.3. Open Anterior Abdominal Approach in Intact Pelvises of Unfixed Cadavers

Two fresh frozen cadavers that had not been previously dissected were used for this study. They were removed from the freezer two days before dissection to fully thaw. On the day of dissection, an anterior abdominal approach was taken to reach the posterior aspect of the pelvis. The pelves were left intact for these dissections (i.e., not hemisectioned). The sacrum, sacral promontory, pubis, ureter and obturator nerves were identified and used as landmarks. Vesical branches located on the posterolateral aspect of the bladder wall were identified and traced back to their respective sacral roots.

### 2.4. Micro-Computerized Tomography (micro-CT)

The vesical branches going to the bladder and ventral rami of the sacral plexus were further examined in three cadavers using micro-CT methods. The vesical branches were removed en bloc with the sacral roots from which they originated by careful dissection. Extraneural connective tissue and fat were not dissected off the nerves to preserve small branches and connections. This tissue block (an average of 17 cm in length and 3–4 cm in width) was first placed into 10% buffered formalin solution for 2–3 days before being placed in Lugol’s iodine stock stain solution (catalog # 26658-04, Electron Microscopy Sciences, Hatfield, PA, USA) for approximately 5 days since that incubation period has been shown as necessary to achieve optimal nerve fascicular contrast for micro-CT visualization of nerves (i.e., to render them radio-opaque). On the day of the micro-CT scan, the tissue block with nerves was removed from the container and rinsed with phosphate-buffered saline to remove excess Lugol solution. The tissue block was then sealed in a layer of parafilm, with a moist piece of gauze at the end of the tissue block and then a layer of cling film wrapped tightly to seal the tissue block. This was performed so the tissue retained moisture during the scan to avoid tissue shrinkage.

The wrapped nerve was placed in a styrofoam cylinder and inserted into a Bruker Skyscan micro-CT 1176 in vivo micro computerized (micro-CT) system (Bruker, Billerica, MA, USA), which has a large format 12-megapixel X-ray camera, and an image field width of 6.8 cm, using the following settings: an image pixel resolution size of 8.92 μm, Al 0.5 mm filter, the voltage of 50 kV, current of 401 μA, rotation step of 0.50°, frame averaging of 5, with the geometrical correction, flat field correction, and median filtering options set to “ON”. The tissue blocks were typically scanned by the system as eight to nine connected segments due to the length of the tissue block (up to 17 cm). The time to scan each individual segment was approximately 54 min; the total scanning time for the entire tissue block was approximately 8 h. During the reconstruction of the images using cone-beam reconstruction software based on the Feldkamp algorithm (Skyscan NRecon, version 2.2.06), a ring artifact correction of 5, a beam hardening correction of 40%, and a smoothing kernel (Gaussian) of 2 was applied to all samples, as was automatic post-alignment, matching and fusion (since there were eight to nine segments to be merged). This process yielded at least 18,000 tomographic sections per tissue block, with a total reconstruction time of 1.6 s per slice. Reconstructed cross-sectional image slices were saved as bmp files, which were visualized using a 3D volume-rendering program (Bruker CTVox program, version 3.3.0). This program allowed visualization of the reconstructed cross-sections in 3D and even virtual “sectioning”. This allowed us to carefully track the vesical branches back to their sacral ventral root origins for confirmation of gross anatomical dissections when needed.

### 2.5. Statistics 

Descriptive statistics were performed using Microsoft Excel. The mean ± standard deviation (SD), mode (the number that occurs most often in the data set), and range are provided, as appropriate for the data. All raw data are provided in Tables and Supplemental Tables.

## 3. Results

### 3.1. Sacral Origin of Nerve Branches to the Bladder 

**Figure 1:**
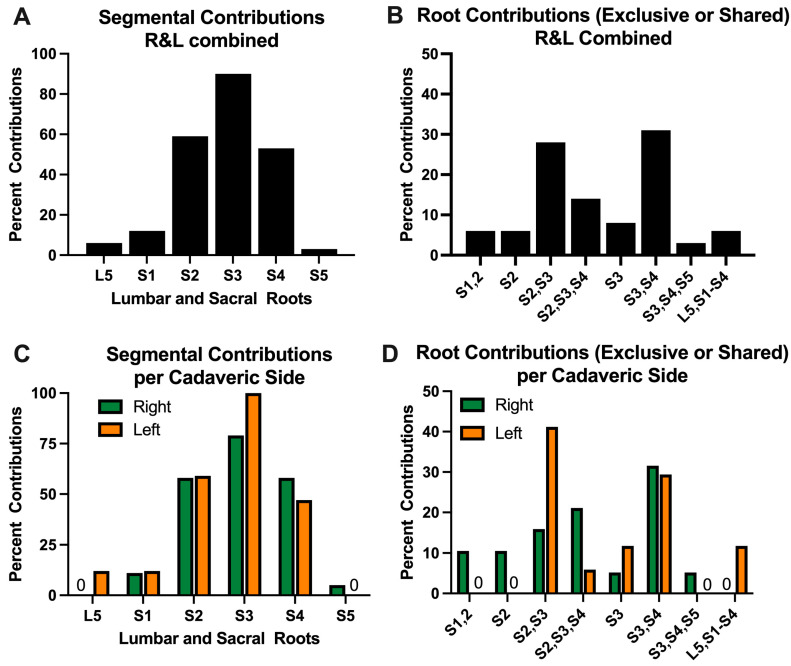
Percent individual or exclusive/shared contributions of lumbar and sacral (L5–S5) ventral rami contributions to the bladder. (**A**) Segmental contributions from each individual segment, with right and left side data combined. (**B**) Root contributions to the bladder (exclusive or shared), with right and left side data combined. (**C**) Segmental Contributions from each individual segment, separated by left vs. right pelvic plexus. (**D**) Root contributions to the bladder (exclusive or shared), separated by left vs. right pelvic plexus. “0” denotes no contribution. Most of the input was from S2, S3, and S4. Raw data is shown in Table 1.

Vesical branches of the inferior hypogastric plexus, and their ventral rami contributions, were examined in 36 pelvic sides of 22 human cadavers (Figure 1A–D and Table 1, which shows raw data). When individual segmental contributions were considered (with right and left side data combined), we observed, in order of primary contributions, that S3 in 90%, S2 contributed to bladder innervation in 59% of hemisected pelvises, and S4 in 53% (Figure 1A, Table 1). L5 provided contributions in 6% of hemisected pelvises, S1 in 12% and S5 in 3. When exclusive or shared root contributions were considered (with right and left side data combined), we observed that sacral origins of these branches were mainly from S3 and S4 (31%), from S2 and S3 (28%), and S2 through S4 (14%), although rarer contributions from either S1 and S2 (6%) or L5, S1 and S5 were also observed in a few cadavers (6%), as were contributions from exclusively S2 or S3 (Figure 1B, Table 1).

### 3.2. Symmetry of Spinal Ventral Contributions to the Bladder

Of the 14 cadavers in which the ventral rami contributions were examined bilaterally, symmetrical right- and left-sided contributions were seen in only four of the cadavers (4/14; 29%; Table 1). Side-to-side asymmetry was observed in 10 of 14 cadavers examined bilaterally (71%). There was contribution overlap in at least one ventral ramus in each of the 14 cadavers (100%). Figure 1C depicts the asymmetry when individual segmental contributions are considered, and the right and left sides are separated (graphical depiction of data from Table 1). S2, S3 and S4 are the primary contributors to bladder innervation. When exclusive or shared root contributions were considered, and the right and left sides were separated, more variability was observed on the right side versus the left (Figure 1D; see Table 1 for raw data).

### 3.3. Representative Examples of Spinal Ventral Contributions to the Bladder

**Figure 2:**
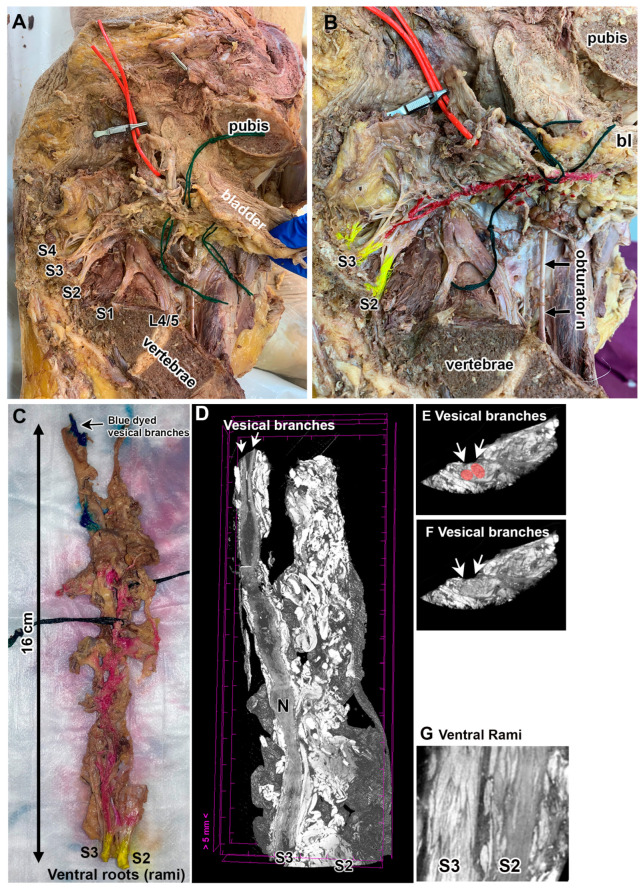
Example of S3 ventral ramus contributions to the bladder. (**A**) A hemisected pelvis of a fixed cadaver, shown looking from the midline (vertebrae) laterally (ureter is marked by a red vessel loop). (**B**) Potential sacral ventral rami contributions are labeled with a yellow tissue dye (S2, S3 and S4 are labeled, although only S2 and S3 appeared to be contributors at this point in the dissection). Their contributions to the vesical branches are grossly marked with a dark red tissue dye for later visualization after removal as a tissue block. (**C**) Tissue block with S2 and S3 ventral rami and vesical branches with adjacent fat, blood vessels and connective tissues. (**D**) Micro-CT was used to scan the entire Lugol-stained tissue block. Longitudinally oriented 3D image shown. (**E**,**F**) Cross-sectional images from the vesical nerve ends of the tissue block. Vesical branches are indicated in both panels with arrows; vesical branches are highlighted with pink in panel (**E**), and are indicated with arrows in both panels (**E**,**F**). (**G**) Close-up of the ventral rami at the proximal spinal cord end from another image. Both S2 and S3 ventral roots are shown. As shown in panel (**E**), the S2 root could not be traced to the vesical branches.

Figure 2 shows a representative example of a contribution from the S3 ventral rami alone. Both gross anatomical dissections and micro-CT imaging methods were used to confirm this contribution. In Figure 2A, a hemisected pelvis is shown in which the bladder is viewed looking from the midline (the cut pubis and sacrum are labeled as landmarks) laterally (the ureter is demarcated by a red vessel loop). While it was clear that S3 contributed to the vesical branches, it was less clear if S2 also contributed. Therefore, this was one of three samples chosen for further micro-CT confirmation. As shown in Figure 2B, potential sacral ventral rami contributions are labeled with a yellow tissue dye for later visualization when removed as a tissue block. Their contributions to the vesical branches of the bladder were traced using careful dissection before marking their general path using a dark red tissue dye for later visualization after the removal of the tissues from the pelvis (Figure 2B). The tissue block with the S2 and S3 sacral ventral rami and vesical branches were removed en bloc, with the adjacent fat, blood vessel and connective tissues left intact (Figure 2C). The tissue block was immersed in Lugol (an iodine stain solution) for 5 days. MicroCT images revealed two vesical branches (Figure 2D–F).

Three-dimensional visualization and examination of the reconstructed micro-CT images revealed that the vesical branches of the bladder arose from only the S3 ventral ramus (Figure 2D). No contribution to a vesical branch could be visualized from the S2 ventral ramus (Figure 2D). As shown in Figure 2D,E, two vesical branches to the bladder could be traced back to the S3 ventral ramus. Figure 2F verifies that both S2 and S3 rami were located in this tissue block.

In other cadavers, the ventral root contributions to the bladder were more typically from two or more ventral rami in combination (Table 1). These ventral rami contributions combined with one another in the following frequencies: S2–S3 (10/36; 28%), S3–S4 (11/36; 31%), and S2–S4 (5/36; 14%). Two cadavers had input from L5 and all sacral segments (7%). In the 17 left sides analyzed, 100% of cadavers had vesical branches originating from S3, while the right side had more variability (Table 1). In most cases, the contributions could be traced by careful dissection alone.

**Figure 3:**
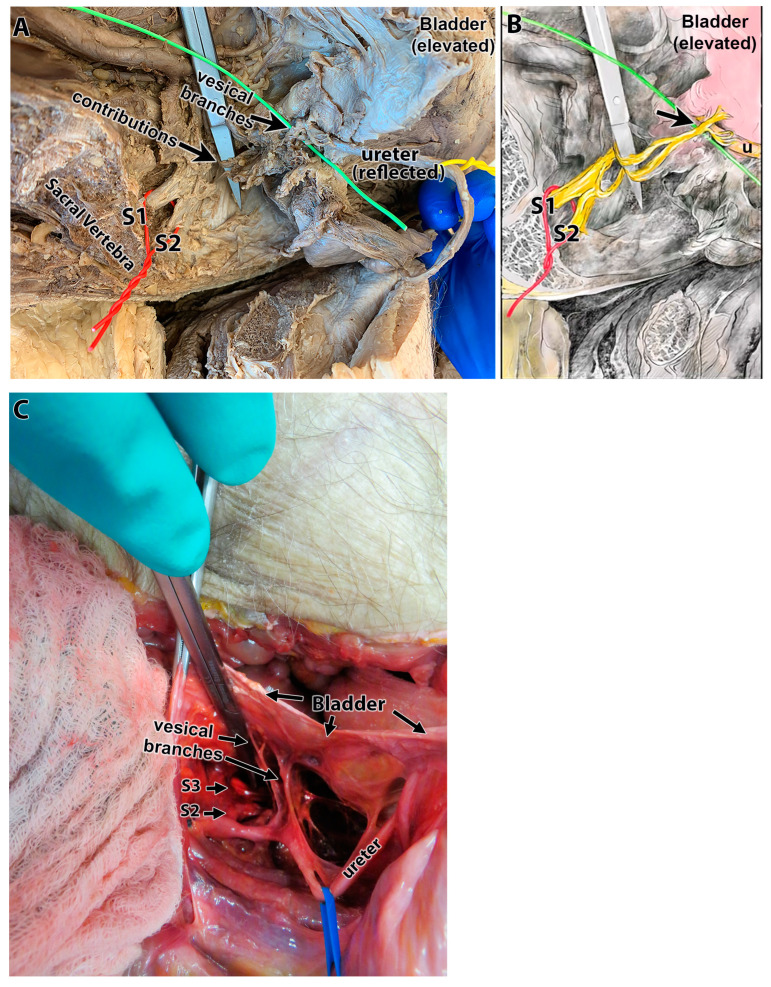
Examples of S1 and S2, and S2 and S3, ventral rami contributions to the bladder in two different cadavers. (**A**) The hemisected pelvis of a fixed cadaver in which ventral rami from both S1 and S2 contributed branches to the bladder (a red wire surrounds these roots). Scissor tips show nerves passing to the vesical branches of the pelvic nerve (overlying the green wire and indicated with an arrow). The ureter is delineated by a yellow wire. (**B**) A diagram of panel (**A**) in which the sacral contributions are colored yellow and the bladder as pink. (**C**) An intact pelvic region of an unfixed cadaver. The image shows a superior view of a fresh pelvic region opened using an anterior abdominal approach. The outer wall of the bladder is labeled; the ureter is indicated by a blue vessel loop. Vesical branches could be seen arising from both S2 and S3 ventral rami (arrows).

Figure 3A,B show a representative example of fairly equal contributions from S1 and 2 in a hemisected pelvis of a fixed cadaver. The ureter is demarcated by a yellow wire, and the sacral vertebral bodies, ureter and bladder are also labeled in the picture as fiduciary markers. The hemisected pelvis is viewed from the midline (location of vertebrae) and laterally (location of the ureter). Figure 3C shows contributions from S2 and S3 in an intact pelvis from an unfixed cadaver (superior view shown). Vesical branches to the outer bladder wall are indicated, as is the ureter.

**Figure 4:**
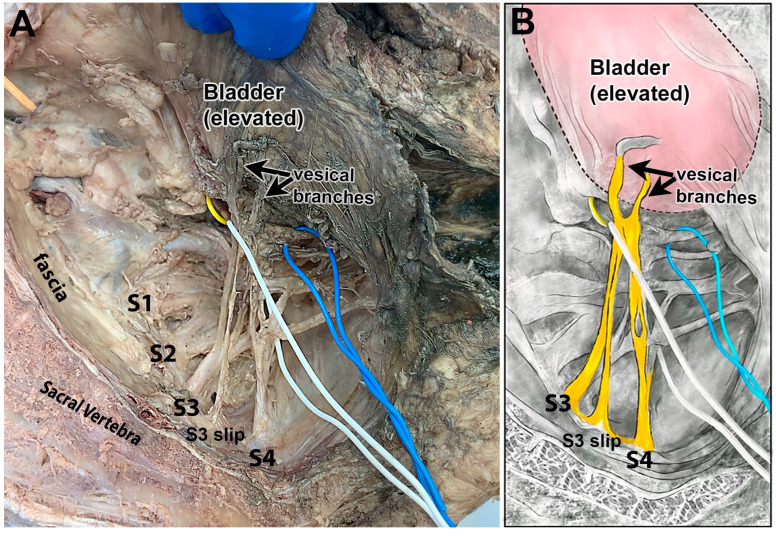
Examples of S3 and S4 ventral rami contributions to the bladder in two different cadavers. (**A**) The hemisected pelvis of a fixed cadaver in which S3 and S4 ventral rami contribute branches to the bladder (encircled by a white wire). S3 had an extra slip arising adjacent to the main root. The ureter, located deep to this view, is encircled with a blue wire. The vesical branches were adjacent to the ureter in this cadaver. (**B**) Diagram of panel A in which the S3 and S4 ventral rami contributions to the bladder are colored yellow, and the bladder is colored pink.

Figure 4A,B show a hemisected pelvis in a fixed cadaver. Vesical branches are shown rising from S3 and S4, with a small additional slip located between the S3 and S4 ventral rami that appeared to be an extra slip from S3. No vesical branches could be traced to the S2 ventral ramus in this cadaver.

**Figure 5:**
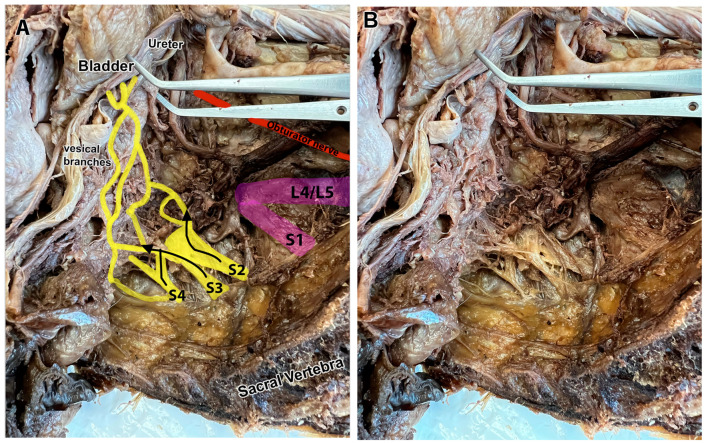
Images of multiple contributions from L5–S4 are more difficult to present as the L5 and S4 branches were quite small in circumference. Example of S2, S3 and S4 combined root contributions to the vesical branches of the bladder (indicated by path of arrows). (**A**) Hemisected right side pelvis of a fixed cadaver depicting S2–S4 ventral root contributions to the bladder, highlighted in yellow. The other sacral roots are indicated in pink, and the obturator nerve is highlighted in red. The forceps were used to elevate the bladder for this view. (**B**) The same image as in Panel (**A**) is shown unlabeled.

Lastly, Figure 5A,B show a hemisected pelvis in a fixed cadaver with contributions from multiple ventral rami, specifically from S2, S3, and S4.

## 4. Discussion

We sought to examine which sacral ventral rami contributes nerve branches to the bladder in human cadavers and the extent of asymmetrical sidedness. Twenty-two human cadavers were dissected, and 44 pelvic sides were examined, although eight were excluded because of damaged structures (leaving 36 pelvic sides). After careful dissection, vesical branches and their sacral ventral rami contributions were traced to the posterolateral aspect of the bladder. Vesical branches were typically found either directly superior or inferior to the ureter, matching prior cadaveric study findings [16]. The ureter, obturator nerve, and sacral prominence were identified during dissection due to their significance as landmarks of surgical importance [28]. Here, the most common contributor to the vesical nerves was the S3 ventral rami, which contributed 100% of the time on the left side and 79% on the right side, while S4 contributed 53% of the time. Sacral ventral rami contributions to vesical nerves were also observed from several combinations of ventral rami: S2 and S3 (28%), S3 and S4 (28%), S2–S4, 14%, L5 in combination with S1–S4 (6%), S1 and S2 (6%), and S3–S5 (3%). Side-to-side asymmetry was observed in 10 of 14 cadavers examined bilaterally (71%).

Living with the consequences of urinary dysfunction after tSCI, colorectal cancer, sacral tumors, or prostatic/gynecological surgeries can have a dramatic impact on one’s quality of life and mental health. Individuals consistently report that bladder function is of high priority to their quality of life [2,9]. As a result, a better understanding of the anatomy of the sacral plexus and its contributions to the bladder will allow for expanded therapeutic interventions for patients. This understanding is crucial to furthering neurourology and techniques for reinnervation of the bladder.

Historically, several studies have described sacral spinal contributions to the pelvic splanchnic nerves in humans as consistently from the S2, S3, and S4 roots/ventral rami [29,30]. Clinical practice has demonstrated variability in sacral root contributions to nerves innervating the bladder between patients [7,17,31]. One study of 40 patients found varying combinations of S2, S3, and S4 sacral contributions (Table 2) [7]. Although there was variability, the S3 root was the most frequently occurring contribution (90%), followed by S2 and S4 on both the right and left sides. However, the authors examined only potential contributions arising from sacral roots, not considering the possibility of lumbar root contributions. This differed from our study in which 6% of individuals had L5 contributions to nerves innervating the bladder. We postulate that differing methodologies contributed to these differences, with Carlucci and colleagues group using neurostimulation methods, while we used cadaveric dissections. The L5 contributions may not have been stimulated in the Carlucci or may be of such low amplitude that they were not detected. Baeder and colleagues reported sex differences, with females showing sacral contributions to pelvic splanchnics from S3 (52%), S4 (32%), S2 (12%), and S5 (4%), while males had sacral contributions to pelvic splanchnics from S3 (60%), S4 (37%), and S2 (~4%) [24]. While we did not see this level of sex differences, our results agree with most human cadaveric and in vivo electrophysiological data showing that the S3 and S4 roots/ventral rami are the primary contributors to bladder innervation [7,17,23,24,32]; S3 contributed 90% of the time in this study. Our results are also in agreement with others, showing that the fewest contributions to bladder innervation are from S5 and S1 (Table 2) [7,24,32]. Animal studies also show considerable between-subject variation in sacral root contributions to the bladder (Table 2) [24,32,33,34,35,36,37], as well as contributions from lumbar roots [34,36,37]. These data combined suggest that spinal contributions are more extensive and varied than previously thought.

Considerable asymmetry was observed in the sacral bladder contributions in this study and a human electrophysiological study, an important consideration when performing bilateral surgery and neurostimulation therapeutics [38,39,40,41]. We observed side-to-side variability in 10 of 14 cadavers examined bilaterally (71%). Two cadavers (#6 and 10) had small contributions from the L5 root on one side, for example. The earlier mentioned retrospective on electrophysiological studies of 40 patients also found asymmetry in left versus right side sacral contributions in 18% of individuals (Table 2) [7]. Lateralization of bladder innervation has been observed in two different dog studies, with some bladders showing left-sided dominance in spinal contributions, others right-sided, and some bilateral [34,42]. Findings of asymmetry are important when considering spinal cord injuries because the extent of the injury and its impact on bladder function may vary in a patient based on this finding. This variation should also be considered when examining the effectiveness of a urological surgical technique. For example, within-patient asymmetry should be considered when assessing patient eligibility for nerve transfer procedures or functional electrostimulation methods with indwelling neurostimulators. Always choosing only one side for a nerve transfer surgery designed to rescue bladder contractability [11] should be avoided as it might be one reason for poor efficacy [12,43]. Instead, electrophysiological testing of a patient’s bladder innervation laterality should be included in the study plan.

Another implication of these findings applies to a common procedure performed for patients with bladder frequency and urgency: sacral neurostimulation/modulation [13,14,15]. Sacral neuromodulation is performed by placing leads next to the nerves in the third sacral foramen. Stimulation within a set field of parameters results in the long-term reduction of bladder frequency and urgency in approximately 60% of patients [15]. Part of the test procedure for placing a temporary lead is that bilateral leads are placed, and the side with the best result is chosen. However, if a more permanent type of lead is placed for a trial in the operating room, then only one side is chosen. These results suggest that a thorough comparison of the two sides is indicated to determine which side may be dominant. This also applies to percutaneous tibial nerve stimulation, where the sides are assumed to be equally efficacious, and one side is randomly chosen to apply the needle [44]. In addition to laterality, in patients who have an unsuccessful trial with a lead solely placed at S3, there could be patients who would benefit from stimulation of another dermatome, alone or in combination [7].

A novel method used in this study was the use of micro-CT to examine nerve branching within the sacral plexus. The Lugol stain, which renders soft tissues radio-opaque, and then micro-CT imaging provided detailed high-resolution images of the sacral plexus in 3D. This allowed us to trace specific contributions of the sacral ventral rami to vesical nerves innervating the bladder, as seen in Figure 2. Although our use of this type of analysis is preliminary, and this type of analysis, in general, is in its nascent stages, it has the potential to specifically characterize which nerve branches are contributing to end organs.

Limitations to this study include the inclusion of only 22 cadavers. While the number of individual humans is more than reported in most past studies, as shown in Table 2, additional studies are warranted. Yet, the findings of this study would be of benefit to any individuals performing a meta-analysis in the future, as suggested for evidence based anatomy [25].

## 5. Conclusions

It has been previously demonstrated that S3 and S4 ventral roots are commonly involved in bladder contraction. However, additional spinal contributions, as well as the different combinations of sacral roots/ventral rami and additional roots/ventral rami innervating the bladder, have not been extensively researched in humans. There appears to be considerable within-subject and between-subject variability in the sacral (or even L5) contributions to vesical branches of the bladder. This has significant implications for providing a better quality of life for patients with urinary incontinence as well as neuro-urological surgeries, such as during nerve-sparing procedures, nerve transfers for bladder reinnervation, functional electrical stimulation, neuromodulation, and more.

## Figures and Tables

**Table 1:** Spinal root/rami contributions to the bladder (specifically, to vesical branches of inferior hypogastric plexus).

Cadaver Number	Spinal Ventral Rami Contributions to Bladder	Spinal Ventral Rami Contributions to Bladder
1	**S2**	NA
2	**S2, S3**	**S2, S3**
3	**S2**	S2, **S3, S4**
4	S2, **S3**, S4	L5, S1, S2, S3, S4
5	**S3, S4**	**S3**, S4
6	**S2, S3**	**S2, S3**
7	S3, **S4**	**S3**, S4
8	NA	**S2**, S3
9	**S3, S4**	**S2, S3**
10	S2, **S3, S4**	**S3, S4**
11	S2, S3	S3, S4
12	S1, **S2**	NA
13	NA	**S3**
14	**S3**	S2, **S3**
15	NA	L5, S1, S2, S3, **S4**
16	S2, **S3, S4**	NA
17	**S3,** S4	**S2, S3**
18	S2, **S3**, S4	**S3,** S4
19	**S3**, S4	NA
20	S3, **S4, S5**	NA
21	**S3**, S4	**S3**
22	S1, S2	S2, S3
% root contributions per cadaveric side	19 right sides: S1—11% S2—58% S3—79% S4—58% S5—5%	17 left sides: L5—12% S1—12% S2—59% S3—100% S4—47%
% root contributions	36 sides: % Individual Contributions: L5—6%; S1—12%; S2—59%; S3—90%; S4—53%; S5—3% % Shared Contributions: S1 and S2 only—6%; S2 and S3 only—28%; S3 and S4 only—31%; S2 thru S4—14%; S3 thru S5—3%; L5 and S1 thru S4—6%	

NA = not assessed; **Bolded** delineates the most prominent contribution.

**Table 2:** Previously reported sacral root/sacral ventral rami contributions to the bladder.

Number of Cases (Humans)	Injury Type/Methods	Greatest Contributor	Least Contributor	Additional Information	Reference
n = 40	A retrospective study involving 40 patients with complete paraplegia or tetraplegia with a spinal injury above the sacral voiding center, unstable overactive bladder, and failure of rehabilitation techniques; and who underwent surgery requiring an electrophysiological exploration of the sacral roots.	S3 in all cases (100%), and exclusively in 8/40 cases (20%) as the sole contributor	S1 (no bladder response when stimulated)	S2, in 15 cases (40%), although the amplitude was often low. S4, in 24 cases (60%). S5, seven cases (18%). S3 and S4 in 12 cases (30%), S2 and S3 in six cases (15%), S2 + S3 + S4 in five cases (12%), S3 + S4 + S5 in two cases (5%). Side-to-side asymmetry in the response was observed in seven cases (18%).	[7]
n = 2 in which bladder function was tested	Neurogenic lower urinary tract dysfunction; performed neurostimulation of S2 and S3 nerve roots before and after selective neurotomy or neural blockade.	S3, with asymmetry observed (L > R) in one case	S2	In one patient, stimulation of the left S3 ventral root produced a strong bladder contraction (80 cm H_2_O) with no urethral sphincter response. Stimulation of the right S3 ventral root produced a weak bladder response with a strong urethral sphincter pressure.	[18]
n = 5	Patients with hypertonic neurogenic bladder (and in which conservative treatment had failed; bladder had preserved elasticity; obstructive lesions ruled out; uninhibited activity on cystometrogram disappeared after selective sacral nerve block or saddle block; improved micturition after the above blocks without causing incontinence) underwent highly selective rootlet rhizotomy	S3 (85%), S4 (46%)	S2	Case 1: Significant detrusor activity was demonstrated on stimulation of the most medially located fibers of the S2 root during a second operation. In all cases, the fibers innervating bladder detrusor muscle were in the most medial portions of the S3 and S4 roots.	[17]
n = 10	Males with suprasacral SCI underwent identification of different neural roots during somatic-autonomic reflex pathway procedure for neurogenic bladder	S3 (7/10, 70%)	L5 and S1 (no bladder response when stimulated)	Either S2 or S3 could result in bladder detrusor contraction; strongest from S3.	[10]
n = 9	Sacral nerve blocks for males with paraplegia and neurogenic bladders.	S2 and S3	S4 (no response in 4/9 cases)	S2 and S3—6/9 cases (66%) S2–S4—3/9 cases (33%) S1—no response in the one case tested	[27]
n = 11, cadavers	Microdissections of sacral origins of pelvic splanchnics performed in 11 Thiel preserved female cadavers.	S2–S4 identified as origins of pelvic splanchnics (target organ not identified)	S1	S2–S4, typical origin of pelvic splanchnics S1, two specimens (18%) had an additional splanchnic nerve from S1 ventral rami.	[23]
n = approximately 63 human cadavers	Cadavers were dissected using a non-surgical laterocaudodorsal approach to analyze the autonomic innervation of the pelvic nerves. Focused here on findings of inferior hypogastric plexus (IHP).	Females: S3 (52%), S4 (32%); and Males: S3 (60%), S4 (37%); identified as origins of pelvic splanchnics to IHP	Females: S2 (12%), S5 (4%); and Males: S2 (~4%)	Target organ not identified for the pelvic splanchnics to IHP. Exact number of cadavers not stated for the 85 hemipelves in which sacral origins of pelvic splanchnics to the IHP.	[24]

## Data Availability

All data are contained within the article and in Supplemental Materials.

## Supplementary Materials

The following supporting information can be downloaded at: https://www.mdpi.com/article/10.3390/diagnostics15010102/s1, Table S1. Cadaver Demographics.
