# Supplementary material for: Variations and Asymmetry in Sacral Ventral Rami Contributions to the Bladder
Source: Diagnostics (Basel). 2025 Jan 3;15(1):102. doi: 10.3390/diagnostics15010102 (PMC11719823; doi:10.3390/diagnostics15010102)
Supplement: Supplementary file 1 [file diagnostics-15-00102-s001.zip › diagnostics-3331639-supplementary.pdf]

Table S1. Cadaver Demographics

Cadaver #	Age	Gender	Relevant observations
1	> 90	F	Formalin-phenol fixed; **Intact (not hemisected);** Healthy appearing bladder; Ureters of normal size (bilaterally); Focused on right side (tissue was quite hardened from the fixation)
2	> 90	F	Formalin-phenol fixed; **Intact initially before being hemisected;** Healthy appearing bladder; very thin wall at ureteral orifice
3	80	F	Formalin-phenol fixed; **Intact initially before being hemisected;** Healthy appearing bladder; Ureters of normal size (bilaterally)
4	56	F	Formalin-phenol fixed; Hemisected pelvis; Large, healthy bladder; Ureters of normal size (bilaterally), although surrounded by an abundance of fibrotic tissue (presumed endometriosis).
5	Unknown	F	Formalin-phenol fixed; **Intact initially before being hemisected;** Healthy appearing bladder, although thick muscular wall visible; Uterus shifted to left due to golf ball-sized, encapsulated uterine fibroid
6	> 90	F	Formalin-phenol fixed; Hemisected pelvis; Healthy appearing bladder; Ureters of normal size (bilaterally); Prior hysterectomy; diverticula in distal colon
7	78	F	Formalin-phenol fixed; Hemisected pelvis; Bladder slightly distended; both ureters enlarged, with R>L; Multiple small calculi in right ureter (like sand); Large neuroma in pelvic ganglion region on right side
8	89	F	Formalin-phenol fixed; Hemisected pelvis Prior hysterectomy and ileostomy; Healthy appearing bladder; Ureters of normal size (bilaterally); unable to assess right side due to hysterectomy scar tissue
9	Unknown	F	Formalin-phenol fixed; **Intact initially before being hemisected;** Small yet healthy bladder; Ureters of normal size (bilaterally);
10	> 90	F	Formalin-phenol fixed; **Intact initially before being hemisected;** Ureters of normal size (bilaterally); Uterine fibroids; Bladder of normal size.
11	90	F	Formalin-phenol fixed; **Intact (not hemisected);** Healthy appearing bladder; Ureters of normal size (bilaterally)
12	78	M	Formalin-phenol fixed; **Intact (not hemisected);** Healthy appearing bladder; Ureters of normal size (bilaterally)
13	80	M	Formalin-phenol fixed; Hemisected pelvis; Healthy appearing bladder; Left ureter of normal size; No data from right hemipelvis (dissected too aggressively).
14	66	M	Formalin-phenol fixed; Hemisected pelvis; Healthy appearing bladder; Ureters of normal size (bilaterally); Slightly enlarged prostate
15	80	M	Formalin-phenol fixed; **Intact initially before being hemisected;** Healthy appearing bladder; Ureters of normal size (bilaterally);
16	86	M	Formalin-phenol fixed; Hemisected pelvis; Ureters of normal size (bilaterally); Examined right side only due to over dissection in prior anatomy class
17	85	M	Formalin-phenol fixed; **Intact initially before being hemisected;** Ureters of normal size (bilaterally); A large tumor was evident in and around bladder. Ganglion engulfed yet sacral contributions still traceable.
18	74	M	Formalin-phenol fixed; **Intact (not hemisected);** Healthy appearing bladder; Ureters of normal size (bilaterally)
19	72	M	Formalin-phenol fixed; **Intact (not hemisected);** Healthy appearing bladder; Ureters of normal size (bilaterally); enlarged prostate
20	67	M	Formalin-phenol fixed; **Intact (not hemisected);** Healthy appearing bladder; Ureters of normal size (bilaterally)
21	82	M	Unfixed; **Intact (not hemisected);** Healthy appearing bladder; Ureters of normal size (bilaterally)
22	79	M	Unfixed; **Intact (not hemisected);** Healthy appearing bladder; Ureters of normal size (bilaterally)

Abbreviations: F = female; M = male
